# The CRISPRdb database and tools to display CRISPRs and to generate dictionaries of spacers and repeats

**DOI:** 10.1186/1471-2105-8-172

**Published:** 2007-05-23

**Authors:** Ibtissem Grissa, Gilles Vergnaud, Christine Pourcel

**Affiliations:** 1 Univ Paris-Sud, Institut de Génétique et Microbiologie, UMR 8621, Orsay, F-91405, France; CNRS, Orsay, F-91405, France; 2 Centre d'Etudes du Bouchet, 5 rue Lavoisier, 91710 Vert le Petit, France

## Abstract

**Background:**

In Archeae and Bacteria, the repeated elements called CRISPRs for "clustered regularly interspaced short palindromic repeats" are believed to participate in the defence against viruses. Short sequences called spacers are stored in-between repeated elements. In the current model, motifs comprising spacers and repeats may target an invading DNA and lead to its degradation through a proposed mechanism similar to RNA interference. Analysis of intra-species polymorphism shows that new motifs (one spacer and one repeated element) are added in a polarised fashion. Although their principal characteristics have been described, a lot remains to be discovered on the way CRISPRs are created and evolve. As new genome sequences become available it appears necessary to develop automated scanning tools to make available CRISPRs related information and to facilitate additional investigations.

**Description:**

We have produced a program, CRISPRFinder, which identifies CRISPRs and extracts the repeated and unique sequences. Using this software, a database is constructed which is automatically updated monthly from newly released genome sequences. Additional tools were created to allow the alignment of flanking sequences in search for similarities between different loci and to build dictionaries of unique sequences. To date, almost six hundred CRISPRs have been identified in 475 published genomes. Two Archeae out of thirty-seven and about half of Bacteria do not possess a CRISPR. Fine analysis of repeated sequences strongly supports the current view that new motifs are added at one end of the CRISPR adjacent to the putative promoter.

**Conclusion:**

It is hoped that availability of a public database, regularly updated and which can be queried on the web will help in further dissecting and understanding CRISPR structure and flanking sequences evolution. Subsequent analyses of the intra-species CRISPR polymorphism will be facilitated by CRISPRFinder and the dictionary creator. CRISPRdb is accessible at

## Background

Clustered regularly interspaced short palindromic repeats (CRISPRs) have been described in a wide range of prokaryotes, including the majority of Archaea and many Bacteria. They consist in the succession of 24–47 bp repeated sequences (often called direct repeats or DR) separated by unique sequences of a similar length (spacers) [1-4]. *Bona fide *CRISPRs possess at one end a partial DR and at the other end after the last DR a sequence of about 200 bp called the leader [5]. The origin of the spacers is still largely unknown but several recent studies identified some of them as fragments of foreign DNA mostly of viral origin [6-9]. Analysis of a large number of *Yersinia pestis *isolates has shown that these elements are sequentially added in a polarised fashion next to the leader [8]. This suggestion was further confirmed by observations in *Sulfolobus solfataricus *and in *Streptococcus thermophilus *[9,10]. A cluster of genes called *cas *(CRISPR-associated) are often found in the vicinity of CRISPRs [5]. When several CRISPRs with the same DR are present, only one is associated with *cas *genes. The exact number of *cas *genes is not known and apparently varies from one strain to another. However, a core of 4 genes is regularly identified, which appears to encode proteins involved in DNA modification and repair [11]. Phylogenetic studies performed on the CAS proteins suggest that CRISPRs are acquired by horizontal transfer [12,13]. This is consistent with their presence on megaplasmids [12]. CRISPRs are non-coding regions but different observations suggest that they are transcribed into small RNAs (smRNA) possibly from the leader acting as a promoter, and that they might play a role as siRNA (small interfering RNA) to block the entry of foreign sequences [10,11,14].

In order to gain further insight into the organisation and behaviour of CRISPR loci it is necessary to perform extensive analyses of the available sequenced genomes. Several studies have been performed, the most extensive being that made on 370 prokaryotic genomes [12]. However, these studies are static and considering the amount of ongoing sequencing projects they are rapidly becoming obsolete. The TIGRFAM database [15] provides information on CAS associated CRISPR loci but it is not dedicated to CRISPR identification and will not report CRISPR structures devoid of neighbouring *cas *genes.

For the algorithmic detection of CRISPR patterns, several methods were empirically applied previously, making use of REPuter [13,16], PatScan [12,17], TRF [8,18], LUNA [10], PYGRAM [19]. These programs are designed to find repeats and are not especially conceived for CRISPR patterns finding, so they may provide the CRISPR location but do not define accurately the consensus DR. The output of such tools requires significant manual discard to eliminate background, and post-processing to define the consensus DR and the spacers. Recently, a CRISPR dedicated software tool called PILER-CR was described [20]. PILER-CR is based on an elegant algorithm that consists mainly in producing piles meeting the CRISPR properties from local alignments of the query sequence to itself. The software tool has the advantage of being rapidly executed but it sometimes misidentifies the DR boundaries and omits the truncated DR.

Finally, using the available programs, "short" or "quite short" CRISPRs (defined as containing less than three, three or seven spacers [5,12,19]) are not considered.

Since future insights into the evolution of CRISPRs may result from the investigation of these very small CRISPRs, some of which may be newly emerging structures, it is important to facilitate access to this enlarged, but much more difficult to define, group.

We have developed tools to identify CRISPRs, select DR and store spacers into dictionaries, and a database which can be queried online at . The CRISPRdb is automatically updated; in the May 2007 version, 475 published microbial genomes have been processed.

## Construction and content

### Database and software design and implementation

CRISPRdb and associated web services are implemented in Perl version 5.8.8 [21] and take advantage of some BioPerl [22] modules for manipulating sequences. They run on an Apache 2.0 web server [23] with a Linux operating system (debian Sarge 3.1) [24]. The core application consists of two main programs: CRISPRFinder to detect CRISPRs and extract them from a genomic sequence, and Database Tools for downloading prokaryotic genomes from the NCBI ftp site [25], saving CRISPRs and making updates.

The first program is a full command line tool written in-house in Perl. It is used to process published genome sequences and feed the CRISPR database. It can also be run interactively through the web interface for submission and analysis of users sequence data [26].

The second program is a set of Perl scripts. Downloading of genomic sequences, CRISPRs detection and motifs extraction are fully automated.

A web resource is built on top of these programs via PHP [27] and Perl CGI scripts. This preserves platform independence across multiple operating systems and allows the user to interact with the different CRISPR tools programs without computer programming or (shell) scripting skills.

### The CRISPRs database (CRISPRdb)

CRISPRdb is a relational database implemented using mysql 4.1 [28]. It utilizes the CRISPRFinder program to identify putative CRISPRs and additional tests to further screen for the smallest CRISPRs in a polyphasic approach. Indeed the CRISPRFinder program is conceived to authorize the largest number of possible CRISPRs, especially the shortest ones, containing one or two spacers. The main idea of the program is to first find possible CRISPR localizations in a genomic sequence and then check if these regions contain a cluster that possess the characteristics of "obvious" CRISPR, i.e. containing at least three repeats. Finding possible CRISPR localizations is achieved using the Vmatch package to detect maximal repeats [29], that is a repeat that cannot be extended in either direction without incurring a mismatch [16,30]. Reported matches must have a size within 23 to 55 bp with one possible mismatch, and the gap size between two instances of a repeat must be within 25 to 60 bp. The maximal repeats are clustered according to their position in the genome. In each "cluster", the maximal repeat which is the most frequent in the genome being processed is selected and "blasted" against the cluster. Such a maximal repeat is a candidate DR sequence, and when additional candidate DRs are identified, a score is computed to select the DR resulting in the minimum number of mismatches towards its boundaries. This step is probably instrumental to achieve a very precise identification of proper DR consensus compared to other programs. The related matches are then extracted and tested as putative DRs of a CRISPR, so that the first or the last match is allowed to be degenerated with a maximal number of errors equal to half the match length. This allows the efficient identification of the first, often truncated, DR. The other matches must be globally conserved at least to 80%. Finally two filters are added to check the CRISPR candidates' structure. The first one eliminates clusters for which spacers length are not within the range of 0.6× and 2.5× the DR length. In addition, CRISPR candidates with more than 60% of similarity between spacers (or between DR and spacer) are considered as tandem repeats and are eliminated by the second filter. The selected criteria described above imply that the minimal structure of a putative CRISPR detected by CRISPRFinder should consist in at least two successive direct repeats (one spacer) with a maximum of one mismatch. CRISPRs of more than 2 spacers with three or more perfect repeats are considered "confirmed CRISPR" whereas the shorter CRISPRs are considered "questionable".

**Figure 1:**
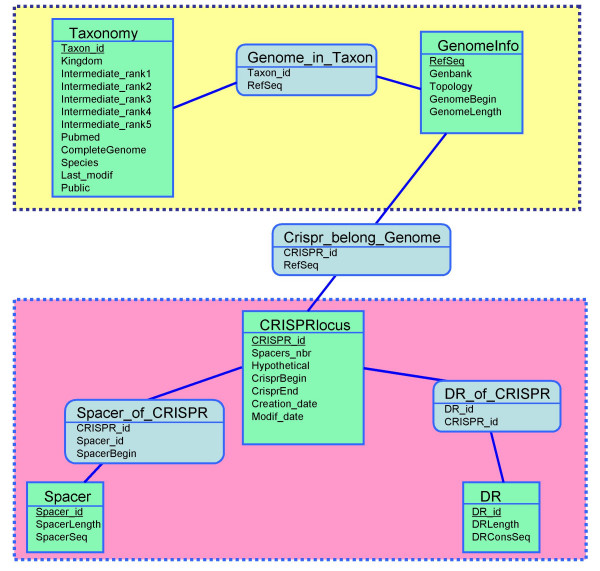
**An entity-relationship diagram for the CRISPR database**. The downloaded data are represented in the yellow box: on the left the taxonomy report information and on the right the "GenomeInfo" report information about species replicons (chromosome or plasmid). The pink box represents tables related to the CRISPR clusters: a table for the cluster locus, a table for the DR consensus and a table for the spacers.

**Figure 2:**
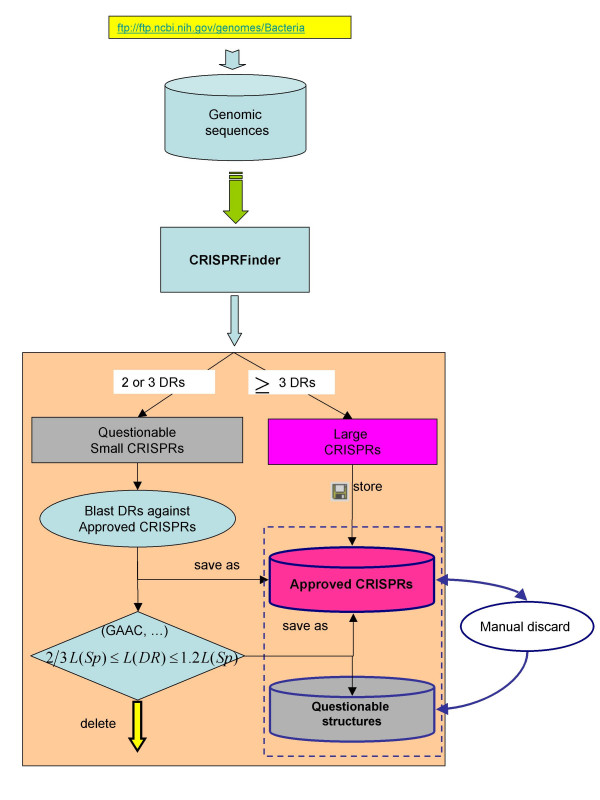
**The database construction: from genomes to CRISPRs**. The first step consists in downloading prokaryotic genomes which are then submitted to the CRISPRFinder program. The detected clusters are divided into two groups: confirmed CRISPRs (>=3DRs) are stored in the database; small questionable clusters (2 or 3 DRs) are analyzed by blasting their conserved region (DR) against the approved DRs; clusters with already identified DRs are added to the CRISPR database. Remaining questionable CRISPRs are analysed for classical flanking nucleotides and spacers length compared to the DR length. Clusters that do not fit these criteria are deleted, the remaining are kept as questionable. Manual discard of some sequences can be performed by the database curator. Colour code: programs are shown in blue, confirmed CRISPRs are in pink and questionable ones are in grey.

Currently, CRISPRdb is composed of 5 tables (Figure 1). For storage in CRISPRdb (Figure 2), several additional tests are applied to the questionable CRISPRs in order to validate a maximum of them. First, a comparison of their DR to previously identified DRs is performed (for example, CRISPR NC_006155_4 in *Yersinia pseudotuberculosis *IP 32953 with 2 spacers has the same DR as CRISPRs NC_006155_6 and NC_006155_7 in the same genome, comprising respectively 4 and 16 motifs; CRISPR NC_003272_3 in Nostoc sp. PCC 7120 with only one spacer, has the same DR as the CRISPR NC_007413_19 of *Anabaena variabilis *ATCC 29413 comprising 33 spacers). Then, a second filter is added to discard some of the non significant short CRISPRs, consisting in a restriction on the spacer allowed length, when the corresponding DR has no classical flanking nucleotides such as GTTT or GAAC.

Authorizing small CRISPR-like structures in the database leads to an important amount of questionable data. Therefore a colour code is being used to differentiate the "confirmed CRISPR" shown in pink to the questionable structures shown in grey. However, and importantly, each time the database is updated, and new genomes are processed, DRs from all questionable structures are rechecked against the updated DR database.

CRISPRs loci are identified from finished microbial genome sequences (as listed by the Genome Online Database [31] and accessed from Genbank) and stored into the database. This procedure is repeated monthly to update the database.

## Utility

### CRISPRdb: construction and content

**Figure 3:**
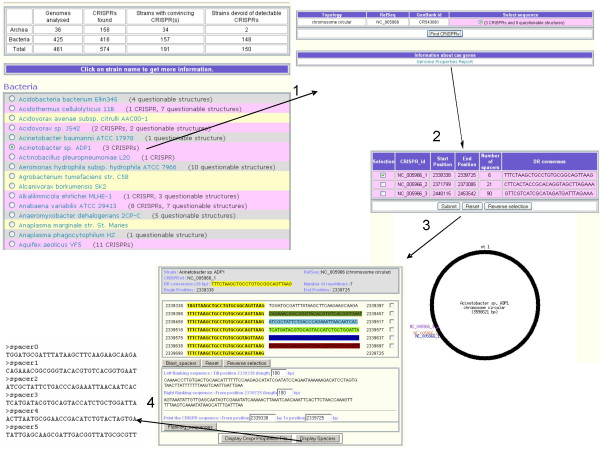
**Screenshots of the CRISPRs web-service**. 1. The opening page of the prokaryotic strains: strains in pink have at least one CRISPR, strains in grey have only questionable CRISPRs and strains in yellow have no CRISPR. 2. General information on the CRISPR clusters and their location. 3. Detailed information on the clusters: DRs are in yellow, spacers are in random colours. 4. Link to the spacers fasta file.

Figure 3 details some of the pages which can be viewed when browsing the database [32]. On the home page (extract, top left) is displayed an alphabetical list of Bacteria and Archaea strains for which genome sequence is published, and a colour code indicates whether a CRISPR has been detected or not: species without a CRISPR are coloured in yellow, and species having at least one CRISPR are coloured in pink. The list can also be sorted according to taxonomic order, or according to database processing date. This last option makes it easy to quickly browse the latest entries. The page which appears after selecting a genome (step 1) indicates how many CRISPRs have been found and on which replicon (chromosome or plasmid) they are located. In the following page (step 2) the CRISPR id is indicated together with its position on the genome, the number of spacers and the consensus DR sequence. Querying a CRISPR locus (step 3) leads to a page containing detailed characteristics together with sequence retrieval tools: the DR consensus is shown in yellow, the spacers are shown in different colours, together with their position in the genome, the flanking sequences and the whole CRISPR locus sequence (using the flanking sequence button). Flanking sequences are displayed with flexible positions that may be modified from the 100 bp default value. Spacers can be automatically compared to public sequences databases using blastn. From this page one can access a flanking sequence CLUSTALW multiple alignment tool (FlankAlign) which is used for defining the presence of a leader and searching for homologous sequences in other genomes.

Furthermore, the ability to upload pre-calculated files (such as a summary of selected CRISPR properties or list of spacers in Fasta format, step 4) makes the tool very flexible, as the output can be analysed with other bioinformatics resources.

### The CRISPR utilities page [33]

**Figure 4:**
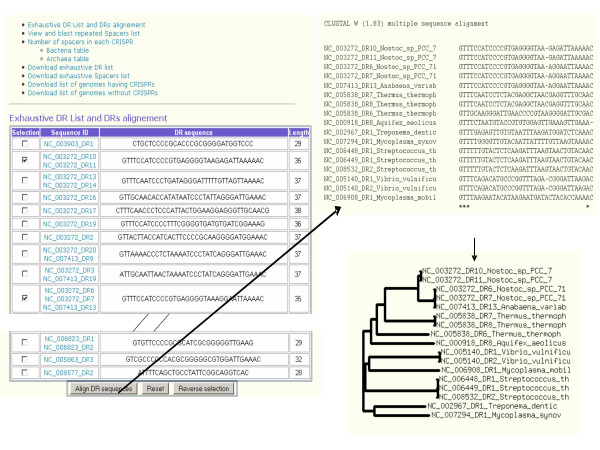
**The DR comparison tool**. Screenshot from the Utilities page showing the list of DRs with an alignment example.

This page provides a global overview of CRISPRs present in the database, focusing on DRs and spacers (Figure 4). Firstly, all identified DRs are listed with their size expressed in base-pairs (bp), and the occurrences in the database of DRs with similar sequences is indicated as shown on the left panel of Figure 4. Selected DRs can be aligned using CLUSTALW and a dendrogram is produced (Figure 4 right panels).

Secondly a list of spacers encountered more than once provides an easy way to identify for instance the relatively rare occurrences of internal duplications within a CRISPR. A BLAST (blastn) can be run using selected spacers against public sequence databases (GenBank, EMBL, DDBJ, PDB) with a cutoff of 0.1 for the E-value and a matching length of at least 70% the queried spacer size. Thirdly, this page provides a classification of CRISPRs according to the number of motifs. The CRISPR id provides the related strain name on mouse-up and links to the page describing the CRISPR properties. Links are also provided to the corresponding pre-computed lists of DRs and spacers which can be downloaded as text files.

### The BLAST CRISPRs page [34]

This page will be of use to try and validate a questionable CRISPR. From this page, a candidate DR region (or spacer) can be compared to all DRs (or spacers) characterised so far from clear-cut CRISPR structures present in the database.

### The Spacers Dictionary Creator page [35]

The analyses of CRISPRs in different strains of a species has shown that polymorphism exists in the number and nature of spacers [8-10,36,37]. This can be used to assess the degree of polymorphism inside the species thus providing additional information for epidemiological analyses. For this reason, it is important to be able to extract spacers from a sequence, and to store them into a database that can be queried when new sequences are produced. Upon submitting CRISPR sequences into the Spacer Dictionary Creator page, spacers are extracted and stored into an Excel file, either predefined or newly created. When a spacer is already present in the dictionary, its number appears in the output whereas a new spacer will be given a new number and will be added into the Excel file.

## Discussion

### Sensitivity and selectivity of CRISPRFinder

To build the CRISPRdb we have used a new program, CRISPRFinder, specifically created to identify CRISPRs. We checked that all the CRISPRs described in the literature were detected with CRISPRFinder and, in addition, we found that CRISPRFinder performs better than other CRISPR finding tools in particular in defining the DR boundaries and in identifying short CRISPRs. Among available programs, we found that PILER_CR is the most efficient. However, in the chromosome of *Aquifex aeolicus *VF5 (NC_000918) for instance, PILER_CR (default parameters: minarray 2, mincons 0.7, minid 0.85) detects 9 CRISPRs, three of which have misidentified DR boundaries and three are missing the truncated DR. In addition, one CRISPR locus is missed because only CRISPRs of at least three repeats are detected. CRISPR NC_000918_6 (one spacer) in the CRISPRdb was not detected by PILER_CR although it has the same DR as CRISPRs NC_000918_1, NC_000918_2, NC_000918_3 and NC_000918_10 containing respectively 5, 4, 3 and 3 repeats). Furthermore, CRISPRFinder is capable of detecting CRISPRs which DRs contain multiple differences such as NC_009009_1 and NC_009009_2 in *Streptococcus sanguinis*. Using the default parameters of PILER_CR no CRISPR was detected in this bacterium. When parameters were changed, only part of the CRISPRs were found. It will be interesting in the future to check whether these exceptional CRISPRs and *cas *genes are functional. Conversely, CRISPRFinder occasionally fails to exclude some false positives. We manually analysed all the CRISPRs identified in the current version of the database and eliminated a few false positive structures, principally tandem repeats with a low internal conservation. We estimate these cases to be less than 1% of "confirmed" CRISPRs.

### Characteristics of CRISPRs

CRISPRdb has been constructed using public domain genome sequences (unpublished sequences can be submitted to CRISPRFinder to detect CRISPRs and extract the spacers). Sixty three percent (63%) of the structures qualifying as CRISPRs using the defined parameters possess 4 or less than 4 spacers. The majority of these are classified as questionable. Their confirmation or exclusion as *bona fide *CRISPR structures will require additional evidence, such as the presence of a DR already described in a CRISPR, the presence of *cas *genes in the vicinity or the search for polymorphism within multiple isolates from the same species.

We have chosen to restrict the definition of CRISPRs to comprise DRs 23 to 55 bp-long and spacers 0.6 to 2.5 the DR size because these sizes are in excess of the range of previously described CRISPRs. These parameters do not exclude CRISPRs also containing a subset of much larger spacers as can be seen in *Methanopyrus kandleri *with spacers 51 to 72 bp-long. There are no clear rules defining the limits of a DR or a spacer and we might be missing currently unknown CRISPRs with characteristics outside of the range currently covered, even if the present rules were deduced from the published investigation by various means of more than 300 genomes. Should such CRISPRs be observed in the future, the database, as designed, can be easily adjusted.

Wide differences are observed among the CRISPRs, in the DR sequence, its size and the size of the spacers. Table 1 summarizes the size distribution observed for DRs. Interestingly, in both Archaea and Bacteria, three well-separated size classes are observed: small DRs (24–25 base-pairs), medium-size (28–30 bp) and large (36–37 bp). The smaller DRs group is more represented in Archaea (42% versus less than 2% for this size class in Bacteria) and curiously it is also where the differences between DR and spacer size are the largest. In *Pyrobaculum aerophilum *7 CRISPRs have a 24 or 25 bp-long DRs whereas the spacer sizes range from 38 to 53 bp. The longer spacers were observed in *Methanopyrus kandleri *which possess 5 CRISPRS with DRs 35 or 36 bp-long and spacers 51 to 72 bp-long, as previously mentioned. In contrast, a remarkably constant spacer length is observed in some bacteria. In *Geobacter sulfureducens *a single CRISPR with a 29 bp DR possess one hundred and thirty eight 32 bp-long spacers and three 33 bp-long spacers. A similar situation is observed in *Mycoplasma mobile *and in *Treponema denticola*. The longest DR presently found is 47 bp-long in the CRISPR of *Bacteroides fragilis*. The associated spacers are either 29 or 30 bp-long. This suggests that the precise mechanisms producing spacers is different from one bacterium or archaeon to another although a common set of CAS proteins is generally associated with all the CRISPRs. The largest CRISPR locus was found in *Verminephrobacter eiseniae *consisting of 245 repeats on one side and 45 repeats on the other side of an IS element (NC_008786_2 and NC_008786_3). The DR is 28 bp-long and the average spacer length is 32 bp. The longest CRISPR previously described was NC_003869_3 from *Thermoanaerobacter tengcongensis *MB4 with 217 repeats.

**Table 1:** Summary of the characteristics and number of CRISPRs.

**DR length**	**Number of CRISPRs (percentage %)**		
	**Bacteria**	**Archaea**	**Total**

47	1 (<1)	0	1 (<1)
38	3 (<1)	0	3 (<1)
37	55 (14.7)	14 (8.9)	69 (13)
36	69 (18.4)	9 (5.7)	78 (14.7)
35	10 (2.7)	1 (<1)	11 (2.6)
34	1 (<1)	0	1 (<1)
33	4 (1)	0	4 (<1)
32	31 (8.3)	1 (<1)	32 (6)
31	6 (1.6)	2 (1.3)	8 (1.5)
30	51 (13.6)	46 (29.1)	97 (18.2)
29	68 (18.1)	9 (5.7)	77 (14.4)
28	67 (17.9)	7 (4.4)	74 (13.9)
27	2 (0.53)	2 (1.7)	4 (<1)
26	1 (0.27)	0	1 (<1)
25	6 (1.6)	37 (23.4)	43 (8)
24	0	30 (19)	30 (5.7)
**Total Number**	375	158	533
**Mean Length**	32	32	32

Only confirmed CRISPRs are counted. The first column shows the DR length. In the second and the third columns are shown the number of clusters having the corresponding DR length in Bacteria and Archaea respectively (the percentage of CRISPR DR having this length is indicated). Only one strain per species is counted. In the last column, the two populations of CRISPRs are merged. The last two lines are respectively the total number of CRISPRs in each category, and the average DR length.

Mojica and col. [4] observed the existence of terminal and inner-inverted repeats in the DR sequence, and Jansen and col. [5] further suggested that the secondary structure might play an essential biological role. A protein binding on one side of the repeat and producing an opening of the opposite side of the DNA structure was described in *Sulfolobus solfataricus *[38] and might be used in the processing of small RNAs [14]. A future development of our work will be the analysis of all the DRs in search for a common secondary structure that might help in understanding the role of the DR.

Inside a species several strains can share a set of spacers, but in a given CRISPR spacers are generally unique except in a few cases where duplications of one to 7 motifs (a DR and a spacer) were observed [33]. Apparently, duplications are more frequently observed in Archaea as described in detail by Lillestol *et al*. [10].

It is important to note that the absence of CRISPR in one strain does not imply that CRISPRs are absent from all the members of the corresponding species. However in some species or genus no CRISPR has been identified yet although a number of strains have been fully sequenced. This is the case for example in *Staphylococcus aureus *and *Burkholderia sp*.

### Multiplication of CRISPR

It is believed that CRISPR and associated genes *cas *can be horizontally transferred between bacteria of different species and possibly between Archaea and Bacteria. This is strongly suggested by comparison of CAS protein sequences, but it does not explain how several CRISPRs with a similar DR can be present in a single genome, only one of which being associated with *cas *genes. The small CRISPRs are particularly interesting in this respect to try and elucidate the mechanism of creation of a new CRISPR and of insertion of new motifs in an existing CRISPR. For example in *Clostridium tetani *among eight CRISPRs possessing 1 to 33 motifs, seven are clustered between position 1570766 and 1595950 (spanning 25.184 bp), five of which with exactly the same DR and two with a derivative (6 different nucleotides out of 30). The leaders of the seven clustered CRISPR aligned over about 150 bp with 80% similarity, *cas *genes are present once between CRISPR 5 and CRISPR 6 and no spacer is in common. It is then most likely that starting from an ancestral complete CRISPR and *cas *genes locus, new CRISPRs have been created not by duplication of the complete complex but rather by the insertion of a minimum structure comprising a leader sequence, a DR, and no spacer, which then grows by adding new motifs. This absence of common spacers even when several CRISPRs are present in a single Bacteria or Archea is also suggesting that gene conversion is not a significant process for new motif acquisition.

### The CRISPR intra-species polymorphism: insight into the mechanism of acquisition of new motifs

We developed the spacer dictionary tool to facilitate the extraction of spacers and their analysis, principally for phylogenetic studies. To better demonstrate the efficiency of this tool we propose a demonstrator based on the sequences of five *Y. pestis *genomes. An initial dictionary was first created from the 26 published spacers, named using the alphabet from "a" to "z" [8]. The CRISPRs of newly sequenced alleles as could be derived from sequencing the locus in a collection of diverse strains can be submitted to the dictionary tool in fasta format. The spacers which were not already present in the dictionary are given a number and they are added sequentially into the dictionary. The alleles are coded in a convenient way using this dictionary.

**Figure 5:**
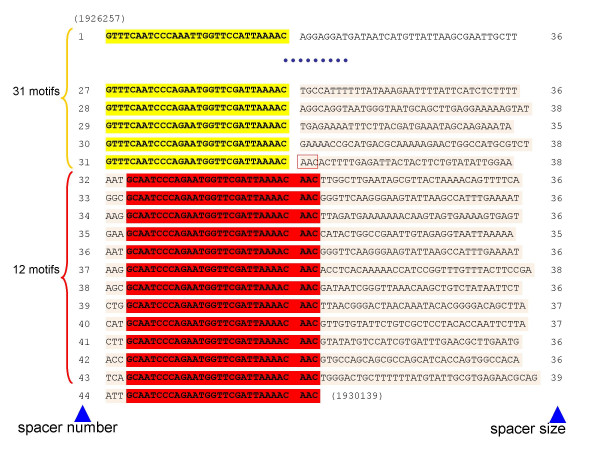
**The first and last 17 motifs of CRISPR NC_007503_3 from *Carboxydothermus hydrogenoformans *Z-2901**. The DRs shared by the two CRISPR loci NC_007503_3 and NC_007503_4 are shown in yellow and the variant DR observed only in NC_007503_3 is in red. CRISPR units (DR + spacer) are numbered on the left and spacers' length is indicated on the right.

In our previous study of three CRISPRs in 180 *Y. pestis *isolates, most of which were genetically very similar, we described the polymorphism at each locus due to different number of motifs [8]. Our observations suggested that one or several motifs could be lost by precise deletion between 2 DRs whereas new motifs were added precisely at the level of the last DR flanking the leader. A similar suggestion was made based upon observations in *S. solfataricus *P1 and in *S. thermophilus *[9,10]. This mechanism is further supported by the analysis of the structure of some CRISPRs in which a first series of motifs containing a particular DR is followed by motifs with a DR differing at a single nucleotide up to the last one near the leader. For example in the CRISPR NC_005085_3 of *Chromobacterium violaceum*, 13 motifs with DR "GTGTTCCCCACG**TG**CGTGGGGATGAACCG" are followed by 6 motifs with DR "GTGTTCCCCACG**CC**CGTGGGGATGAACCG". Another interesting example is found in *Carboxydothermus hydrogenoformans *where two CRISPRs, NC_007503_3 and NC_007503_4 (59 and 84 spacers respectively) share the same 30bp-DR, although in one of them the last 13 repeats adjacent to the leader have a modified DR. The first three bases of the DR are absent whereas the three bases AAC are added to the other end to produce a modified DR (Figure 5). This suggests that at some point the last DR plus 3 bases of a newly added spacer were duplicated to create a new DR which then served as a matrix for subsequent duplications. Alternatively, the AAC addition could be the result of some stuttering since the initial DR ends by AAAAC (and the modified DR by AAAACAAC). These observations are in favour of the model of polarised sequential insertion of new motifs by duplication of the last DR and insertion of a new spacer [8,10], rather than random insertion by homologous recombination as proposed by Makarova *et al*. [11]. If the newly copied last DR contains a mutation, compatible with CRISPR metabolism, then this mutation will be copied in all subsequent motif acquisitions.

### Future developments

Further development of our software will include new parameters to analyse genomes for which only questionable structures were detected. An additional aspect will be the identification of minimum CRISPRs structure, devoid of spacers and comprising only a DR and leader.

## Conclusion

The described software and database are exclusively devoted to the identification and the analysis of CRISPRs structures, *i.e*. the succession of motifs made up of DRs and spacers. A database for *cas *gene identification has been developed by TIGR [15]. We have added a link to this web page in order to search for the presence of *cas *genes in the vicinity of a CRISPR.

CRISPRs are fascinating structures, which conceal complex biological mechanisms to account for their transfer, evolution and behaviour. They have probably played an important role in the evolution of Archaea and Bacteria by providing a defence mechanism against foreign DNA. A lot remains to be discovered, and this necessitates the possibility to rapidly investigate newly sequenced genomes, and to be able to easily browse across many different species. The CRISPRdb and associated web service provides all the necessary tools to decipher the organisation of these structures. Several studies have shown that when an origin can be found for a spacer, it is most frequently a virus or a plasmid sequence. Thus the spacer database will serve as a repository of sequences of probable viral or plasmid origin. Finally the intra-species polymorphism of CRISPRs and their evolution mode (organised acquisition and loss of motifs) make them interesting tools for epidemiological studies. The possibility exists that a given spacer be added twice independently into a CRISPR, which could hamper its use for phylogenetic studies. However the polarized addition of motifs, and limited events of recombination insure that their order should be preserved. In *Y. pestis *we believe that they could be used to investigate ancient DNAs (Vergnaud et al. in press).

## Availability and requirements

The resource described here is accessible with no restrictions, except for the demand to quote the site [32] (see Creative Commons license on the site).

## Authors' contributions

GV and CP designed the study. IG developed the programs and database, and ran initial tests. Additional tests were done by IG, GV and CP together with collaborators. CP, GV and IG wrote the manuscript. All authors read and approved the final manuscript.

## References

[B1] Nakata A, Amemura M, Makino K (1989). Unusual nucleotide arrangement with repeated sequences in the *Escherichia coli* K-12 chromosome. J Bacteriol.

[B2] Groenen PM, Bunschoten AE, van Soolingen D, van Embden JD (1993). Nature of DNA polymorphism in the direct repeat cluster of *Mycobacterium tuberculosis*; application for strain differentiation by a novel typing method. Mol Microbiol.

[B3] Mojica FJ, Ferrer C, Juez G, Rodriguez-Valera F (1995). Long stretches of short tandem repeats are present in the largest replicons of the Archaea *Haloferax mediterranei* and *Haloferax volcanii* and could be involved in replicon partitioning. Mol Microbiol.

[B4] Mojica FJ, Diez-Villasenor C, Soria E, Juez G (2000). Biological significance of a family of regularly spaced repeats in the genomes of Archaea, Bacteria and mitochondria. Mol Microbiol.

[B5] Jansen R, Embden JD, Gaastra W, Schouls LM (2002). Identification of genes that are associated with DNA repeats in prokaryotes. Mol Microbiol.

[B6] Bolotin A, Quinquis B, Sorokin A, Ehrlich SD (2005). Clustered regularly interspaced short palindrome repeats (CRISPRs) have spacers of extrachromosomal origin. Microbiology.

[B7] Mojica FJ, Diez-Villasenor C, Garcia-Martinez J, Soria E (2005). Intervening sequences of regularly spaced prokaryotic repeats derive from foreign genetic elements. J Mol Evol.

[B8] Pourcel C, Salvignol G, Vergnaud G (2005). CRISPR elements in *Yersinia pestis* acquire new repeats by preferential uptake of bacteriophage DNA, and provide additional tools for evolutionary studies. Microbiology.

[B9] Barrangou R, Fremaux C, Deveau H, Richards M, Boyaval P, Moineau S, Romero D, Horvath P (2007). CRISPR provides acquired resistance against viruses in prokaryotes. Science.

[B10] Lillestol RK, Redder P, Garrett RA, Brugger K (2006). A putative viral defence mechanism in archaeal cells. Archaea.

[B11] Makarova KS, Grishin NV, Shabalina SA, Wolf YI, Koonin EV (2006). A putative RNA-interference-based immune system in prokaryotes: computational analysis of the predicted enzymatic machinery, functional analogies with eukaryotic RNAi, and hypothetical mechanisms of action. Biol Direct.

[B12] Godde JS, Bickerton A (2006). The repetitive DNA elements called CRISPRs and their associated genes: evidence of horizontal transfer among prokaryotes. J Mol Evol.

[B13] Haft DH, Selengut J, Mongodin EF, Nelson KE (2005). A Guild of 45 CRISPR-Associated (Cas) Protein Families and Multiple CRISPR/Cas Subtypes Exist in Prokaryotic Genomes. PLoS Comput Biol.

[B14] Tang TH, Bachellerie JP, Rozhdestvensky T, Bortolin ML, Huber H, Drungowski M, Elge T, Brosius J, Huttenhofer A (2002). Identification of 86 candidates for small non-messenger RNAs from the archaeon *Archaeoglobus fulgidus*. Proc Natl Acad Sci U S A.

[B15] The TIGRFAM page. http://www.tigr.org/TIGRFAMs/.

[B16] Kurtz S, Choudhuri JV, Ohlebusch E, Schleiermacher C, Stoye J, Giegerich R (2001). REPuter: the manifold applications of repeat analysis on a genomic scale. Nucleic Acids Res.

[B17] Jansen R, van Embden JD, Gaastra W, Schouls LM (2002). Identification of a novel family of sequence repeats among prokaryotes. Omics.

[B18] Benson G (1999). Tandem repeats finder: a program to analyze DNA sequences.. Nucleic Acids Res.

[B19] Durand P, Mahe F, Valin AS, Nicolas J (2006). Browsing repeats in genomes: Pygram and an application to non-coding region analysis. BMC Bioinformatics.

[B20] Edgar RC (2007). PILER-CR: fast and accurate identification of CRISPR repeats. BMC Bioinformatics.

[B21] The Perl directory. http://www.perl.org/.

[B22] BioPerl. http://www.bioperl.org/.

[B23] The Apache Software Foundation. http://www.apache.org/.

[B24] Debian. http://www.debian.org/.

[B25] The NCBI ftp site for Bacterial and Archaeal genome sequences. ftp://ftp.ncbi.nih.gov/genomes/Bacteria.

[B26] The CRISPRFinder. http://crispr.u-psud.fr/Server/CRISPRfinder.php.

[B27] PHP. http://www.php.net/.

[B28] MySQL. http://www.mysql.com/.

[B29] Vmatch. http://www.vmatch.de/.

[B30] Abouelhoda M, Kurtz S, Ohlebusch E (2004). Replacing suffix trees with enhanced suffix arrays.. Journal of Discrete Algorithms.

[B31] Liolios K, Tavernarakis N, Hugenholtz P, Kyrpides NC (2006). The Genomes On Line Database (GOLD) v.2: a monitor of genome projects worldwide. Nucleic Acids Res.

[B32] The CRISPR database. http://crispr.u-psud.fr.

[B33] CRISPRUtilities. http://crispr.u-psud.fr/crispr/CRISPRUtilitiesPage.html.

[B34] BLAST CRISPRs. http://crispr.u-psud.fr/crispr/BLAST/CRISPRsBlast.php.

[B35] The CRISPR spacers dictionary. http://crispr.u-psud.fr/crispr/MultipleAnalysis/CRISPRdetector.php.

[B36] Schouls LM, Reulen S, Duim B, Wagenaar JA, Willems RJ, Dingle KE, Colles FM, Van Embden JD (2003). Comparative genotyping of *Campylobacter jejuni* by amplified fragment length polymorphism, multilocus sequence typing, and short repeat sequencing: strain diversity, host range, and recombination. J Clin Microbiol.

[B37] Hoe N, Nakashima K, Grigsby D, Pan X, Dou SJ, Naidich S, Garcia M, Kahn E, Bergmire-Sweat D, Musser JM (1999). Rapid molecular genetic subtyping of serotype M1 group A *Streptococcus *strains. Emerg Infect Dis.

[B38] Peng X, Brugger K, Shen B, Chen L, She Q, Garrett RA (2003). Genus-specific protein binding to the large clusters of DNA repeats (short regularly spaced repeats) present in Sulfolobus genomes. J Bacteriol.

